# Long-Term Rock Phosphate Fertilization Impacts the Microbial Communities of Maize Rhizosphere

**DOI:** 10.3389/fmicb.2017.01266

**Published:** 2017-07-11

**Authors:** Ubiana C. Silva, Julliane D. Medeiros, Laura R. Leite, Daniel K. Morais, Sara Cuadros-Orellana, Christiane A. Oliveira, Ubiraci G. de Paula Lana, Eliane A. Gomes, Vera L. Dos Santos

**Affiliations:** ^1^Microbiology Department, Universidade Federal de Minas Gerais Belo Horizonte, Brazil; ^2^Biosystems Informatics and Genomics Group, René Rachou Research Center, Fiocruz Belo Horizonte, Brazil; ^3^Microbiology Institute, Czech Academy of Sciences – CAS Prague, Czechia; ^4^Centro de Biotecnología de los Recursos Naturales, Facultad de Ciencias Agrarias y Forestales, Universidad Católica del Maule Talca, Chile; ^5^Embrapa Maize and Sorghum Sete Lagoas, Brazil

**Keywords:** microbial community, maize rhizosphere, rock phosphate

## Abstract

Phosphate fertilization is a common practice in agriculture worldwide, and several commercial products are widely used. Triple superphosphate (TSP) is an excellent soluble phosphorus (P) source. However, its high cost of production makes the long-term use of crude rock phosphate (RP) a more attractive alternative in developing countries, albeit its influence on plant-associated microbiota remains unclear. Here, we compared long-term effects of TSP and RP fertilization on the structure of maize rhizosphere microbial community using next generation sequencing. Proteobacteria were dominant in all conditions, whereas Oxalobacteraceae (mainly *Massilia* and *Herbaspirillum*) was enriched in the RP-amended soil. *Klebsiella* was the second most abundant taxon in the RP-treated soil. *Burkholderia* sp. and *Bacillus* sp. were enriched in the RP-amended soil when compared to the TSP-treated soil. Regarding fungi, Glomeromycota showed highest abundance in RP-amended soils, and the main genera were *Scutellospora* and *Racocetra*. These taxa are already described as important for P solubilization/acquisition in RP-fertilized soil. Maize grown on TSP and RP-treated soil presented similar productivity, and a positive correlation was detected for P content and the microbial community of the soils. The results suggest changes of the microbial community composition associated to the type of phosphate fertilization. Whilst it is not possible to establish causality relations, our data highlights a few candidate taxa that could be involved in RP solubilization and plant growth promotion. Moreover, this can represent a shorter path for further studies aiming the isolation and validation of the taxa described here concerning P release on the soil plant system and their use as bioinoculants.

## Introduction

Maize (*Zea mays* L.) is one of the main cereals produced in the world, with approximately 900 million tons produced annually (United State Department of Agricultural, 2017). Phosphorus (P) is an essential nutrient for this crop, especially in the flowering and grain filling stages (Vasconcellos et al., 2000) and to ensure this production, millions of tons of P fertilizer are added to soils each year (Koppelaar and Weikard, 2013). However, this overuse of P fertilizers raises the cost of production and can exerts negative impacts on aquatic environment by the eutrophication of surface water (Dodds et al., 2009). In addition, phosphate reserves are a finite resource that are predicted to be depleted in the next few centuries (Cordell and White, 2011). This can become a limiting factor in global food production. Research efforts have been directed to the use of rock phosphate (RP) as P fertilizers as they have lower costs, are agronomically more useful and environmentally more feasible than soluble P. Although RP has a lower reactivity than commercial fertilizers for direct application on the soil, P availability from these rocks can be increased over years of cultivation, for instance, due to the action of the soil microbiota (Coutinho et al., 1991). Actually, P-solubilizer microorganisms have been described at this niche (Whitelaw, 1999; Linu et al., 2009; Silva et al., 2014; Matthews and Adzahar, 2016). Organic acid production has been pointed as the main factor involved in RP microbial solubilization (Rashid et al., 2004; Mendes et al., 2014). Nevertheless, other mechanisms, such as the release of H^+^ ions during NH_4_^+^ assimilation or metabolic reactions that trigger proton excretion such as cellular respiration, can also contribute to P solubilization (Illmer and Schinner, 1995). Furthermore, microorganisms at the rhizosphere can increase P supply by hydrolysis of organic P through the action of phosphatases, especially phytases (Richardson and Simpson, 2011). Important factors for increasing plant P acquisition includes roots architecture alteration in many plant families like root cluster formation, proliferation and increase of lateral length, a higher number of root hairs, and a reduced length and thickness of the primary root (Hammond et al., 2009; Hunter et al., 2014). All these processes have a strong influence on the microbial communities that colonize the rhizosphere of these plants, and studies suggest that plants can attract P solubilizing microorganisms according to their root structure and the compounds released from exudates (Bolan et al., 1997; Grayston et al., 1998; Broeckling et al., 2008; Guo and Wang, 2009; Hartmann et al., 2009; Chaparro et al., 2012; Hunter et al., 2014).

The impact of RP on rhizosphere microbial diversity is still poorly understood. Recently, culture-independent methods, such as “meta-omics” based on next generation sequencing, have helped to shed some light on the diversity associated to plant–microorganism–soil interactions (Aira et al., 2010; Chhabra et al., 2013; Peiffer et al., 2013; Li et al., 2014). These techniques provide access to a collection of microbial groups related to certain environmental conditions and contribute to the description of non-cultivated taxa by the classic methods of microbiology. Based on the exposed, we used a culture-independent approach to analyze the effect of 3 years of maize fertilization with RP on the microbial community diversity of the rhizosphere. We hypothesized that long-term RP fertilization will drive the selection of efficient P solubilizing or uptake microorganisms to the rhizosphere of maize comparing to conventional use of soluble fertilizers. The results may contribute to understanding of the microbial ecosystem services to the plants. In the future, the use of microbial inoculants able to improve P release/acquisition by plants along with RP fertilization can become a sustainable agriculture practice that will reduce of the environmental impacts besides of the costs production.

## Materials and Methods

### Experimental Design

The field site is located at Embrapa Maize and Sorghum in Sete Lagoas, Minas Gerais, Brazil (19°28′S 44°15′W). The experiment was conducted in an agricultural Oxisol of the Brazilian Savanna biome (Cerrado), classified as a low P soil (Supplementary Table S1). This area was cultivated for 3 years with the 30F35YH hybrid maize (Pioneer, Brazil) on a system of two annual cultivations, one in summer and the other in winter. Each cultivation was conducted using the same P fertilization conditions, namely, soil fertilized with RP (rock phosphate of Araxa), soil with triple superphosphate (TSP, a commercial P fertilizer), and soil without added P. The experimental design was composed of these three types of P fertilization in three replicates. P_2_O_5_ concentration on the control soil was 4 kg/ha and was adjusted to 100 kg/ha on the RP or TSP fertilized soil. Each experimental plot consisted of six rows that were 5 m long and spaced at 70 cm. The useful experimental area consisted of 14 m^2^ of the four central rows of 5 m, eliminating the two border rows.

### Sample Collection for Metataxonomic and Growth Parameters Analysis

Rhizospheric soil was collected at 60 days after sowing (flowering time) from the plants in the summer period of the 3rd year of cultivation. Each triplicate consisted of five randomly chosen plants. Initially, the roots of the plants collected in the field were washed with water under pressure to remove excess soil. Then, approximately 5 g (fresh weight) of the fine roots with adhering rhizosphere soil from each replicate were transferred to 50 mL Falcon tubes with 30 mL of phosphate buffer (per liter: 6.33 g of NaH_2_PO_4_.H_2_O and 16.5 g of Na_2_HPO_4_.7H_2_O). Samples were shaken for 30 s and the roots were transferred to new Falcon tubes containing 30 mL of phosphate buffer. The soil obtained from this procedure corresponded to the first soil fraction. Roots were homogenized in the phosphate buffer again and sonicated at low frequency (50–60 Hz) for 5 min, followed by five cycles of sonication for 30 s and rest for 30 s. Roots were removed and the second fraction of the obtained soil was mixed with the first soil fraction and centrifuged. The pellet obtained after this procedure, considered rhizosphere soil, was frozen in liquid nitrogen and stored at -80°C until DNA extraction.

Subsamples of shoots and grains corresponding to a stand of 45 plants at the end of the experiment (approximately 90 days of culture) were collected for measuring parameters related to plant growth, such as nutrients content, plant biomass and grain productivity. Also, the rhizosphere soil of five plants in three replicates for each treatment was collected at flowering time for determination of P content.

### PCR Amplification and Sequencing

Metagenomic DNA was extracted from rhizosphere soil samples using a Max Power Soil DNA Kit (MO BIO Laboratories, Inc., Carlsbad, United States). DNA was quality-checked using agarose gel electrophoresis and quantified by absorbance at 260 nm on a spectrophotometer (NanoDrop Technologies, Wilmington, DE, United States). The V3–V4 region of the 16S rRNA gene was amplified from DNA using the primer pair 341F and 806R (Klindworth et al., 2013) and the ITS region was amplified using the primer pair ITS3_KYO1F and ITS4_KYO1R (Toju et al., 2012) (Supplementary Table S2). The primers were modified to contain an overhang sequence complementary to the Nextera Index (Illumina, San Diego, CA, United States). A PCR reaction was performed in 25 μL of final volume, which contained, briefly, 12.5 μL of PCR buffer (2× KAPA HiFi HotStart ReadyMix), 5 μM of primer and 30 ng of DNA. PCR amplification was performed using 95°C for 3 min of initial denaturation, followed by 30 cycles at 95°C for 30 s, annealing at 60°C for 30 s and extension at 72°C for 30 s and a final extension at 72°C for 5 min. The amplicons were purified with AMPure XT beads (Beckman Coulter Genomics, Danvers, MA, United States). Then, amplicons were ligated to an index sequence during a second PCR, containing 12.5 μL of PCR buffer (2× KAPA HiFi HotStart ReadyMix), 3 μL of each Nextera XT index, 2.5 μL of the PCR purified product and 7 μL of ultrapure water (25 μL final volume). Amplification was performed at 95°C for 3 min, eight cycles at 95°C for 30 s, 55°C for 30 s, 72°C for 30 s and a final extension at 72°C for 5 min. Amplicons were purified using AMPure XP beads and the size of the libraries was checked using Bioanalyzer DNA 1000 Assay (Agilent, Santa Clara, CA, United States) and quantified using the KK4824 Kapa kit (Biosciences, Woburn, MA, United States). The paired-end (2 × 300 bp) libraries were sequenced using a MiSeq Reagent V3 Kit.

### DNA Sequence Analysis

The DNA sequences were analyzed using a modified version of the pipeline recommended by the Brazilian Microbiome Project (http://www.brmicrobiome.org/; Pylro et al., 2014). Briefly, quality control of the sequence data was performed with Trimmomatic v0.32 (Bolger et al., 2014), with an average Phred score of 15 into a sliding window composed of 4 bp. Then, the sequences were truncated to a size of 400 bp for bacteria and 300 bp for fungi, and unique sequences (singletons) were removed. Chimeric sequences were filtered using USEARCH (Edgar, 2010). Taxonomy was assigned using Greengenes_13_08 for the bacteria database and UNITE_2016_08_04 for the fungi database at 97% similarity and was performed using the QIIME package (Caporaso et al., 2010). To improve the taxonomic assignment of the Oxalobacteraceae and Gigasporaceae families, their sequences were also aligned using the SINA Alignment Service from Silva (Pruesse et al., 2012) and NCBI BLASTn (Altschul et al., 1990) from GenBank, respectively.

The sequence data have been submitted to the GenBank databases under accession number PRJNA379083 for bacterial community and PRJNA379918 for fungal community.

### AMF Community Analysis by T-RFLP

For arbuscular mycorrhizal fungi (AMF) community analysis by terminal restriction fragment length polymorphism (T-RFLP), the 28S rRNA gene was amplified using the general fungal LR1 and FLR2 primers (Van Tuinen et al., 1998; Trouvelot et al., 1999), followed by a nested PCR reaction employing the AMF-specific primers FLR3 (5′ labeled with 6-FAM) and FLR4 (5′ labeled with NED) (Gollotte et al., 2004). The PCR contained 1× reaction buffer, 0.2 μM of each primer, 2.5 mM of MgCl_2_, 0.125 mM of dNTPs, 2.5 U of Taq DNA polymerase (Invitrogen, Carlsbad, CA, United States) and 50 ng of DNA in a final volume of 50 μL. A nested PCR reaction was performed with 2.5 μL of the products from the first PCR reaction in the same conditions reported above. Thermal cycling for all reactions included an initial denaturing step of 95°C for 5 min, 35 cycles consisting of 1 min at 95°C, 1 min at 58°C and 1 min at 72°C, followed by a final extension step of 72°C for 10 min. The amplified fragments were digested using the restriction enzyme *Taq*I (New England Biolabs, Beverly, MA, United States) and were incubated for 6 h at 65°C. To evaluate the generated fragments, 2 μL of the digestion was mixed to 9.8 μL of deionized formamide (Applied Biosystems, Foster City, CA, United States) and 0.2 μL standard ROX 500 (Applied Biosystems). Analysis was performed on the 3500 XL Genetic Analyzer (Applied Biosystems) using the GeneMapper 5.0 software. T-RF profiles were selected by the software if their minimum peak height was above the noise observed, usually above 50 relative fluorescence units. Only peaks between 50 and 500 bp were considered to avoid peaks caused by primer-dimers and to obtain fragments within the linear range of the internal size standard. T-RF length profiles for AMF were loaded into the online T-RFLP processing software T-REX (Culman et al., 2009) for noise filtering and peak alignment.

### Statistical Analysis

Nutrient content, plant biomass and grain productivity of the plants were measured and submitted to variance analysis. Means of different response factors were compared by a Scott Knott test (*p* < 0.05).

T-RFLP data were analyzed using non-metric multidimensional scaling (NMDS) with a Jaccard similarity matrix. Permutational multivariate analysis of variance of the distance matrix was then performed by an Adonis test using the Vegan package in the R program (R Development Core Team, 2008).

Taxonomic classification and alpha and beta diversity analyses were performed using the core diversity pipeline of the QIIME package. Rarefaction curves were constructed based on the observed operational taxonomic units (OTUs) number per reads for bacteria and fungi, and these curves were evaluated by good coverage analysis. To assess the diversity of the samples, we used the coverage estimator Ace, the richness estimator Chao1 and the Shannon and Simpson diversity indices.

Analyses of variance were performed to indices, followed by a Scott Knott test at 5% probability for Ace, Chao1, and Simpson (for bacteria and fungi) and for Shannon (for bacteria). Analysis of variance of the Shannon index for fungi was performed using a Poisson distribution, followed by a contrast test at 5% probability. Additionally, for comparing the OTU number, we used the negative binomial distribution, followed by a contrast test at 5% probability. Variance analysis, followed by a Scott Knott test at 5%, was also used to determine differences at the levels of phyla and families (only for groups with at least 3% relative abundance). Moreover, significance analysis of the relative abundance of the bacterial and fungal taxa was held at a family level (values greater than 0.1%) using the edgeR package. All of the above analysis were performed using R program.

Principal coordinates analysis (PCoA) was performed using the weighted and unweighted UniFrac distance metrics for bacteria and the Bray–Curtis metric for fungi to show the influence of P fertilization on beta diversity. Similarity analysis (ANOSIM) of the clustering was used to evaluate the contribution of the P treatments on microbial communities between samples. In addition, we conducted an NMDS analysis using the Rho metric for bacteria and fungi to assess the sources of variation in the bacteria and fungi community matrices (constructed using OTU number, taxa richness, and family abundance) due to plant biomass, grain yield and P accumulated in the soil using the Past program.

## Results

### P Fertilization Affects the Structure and Composition of the Maize Rhizosphere Microbial Community

Following assembly and quality filtering, a total of 667,222 high-quality reads of the bacterial 16S rRNA gene were obtained from nine samples of rhizospheric soil with an average of 74,136 reads/sample. For fungal amplicon libraries, 2,329,006 high-quality reads were also obtained from the nine samples of rhizospheric soil (an average of 258,778 reads per sample). OTUs having at least 97% sequence similarity with reads deposited in the used databases (Greengenes and Unite) was represented in rarefaction curves within each treatment (**Supplementary Figures S1A,C**). Rarefaction curves tend to an asymptote reaching 0.99 of Good Coverage values (**Supplementary Figures S1B,D**), indicating that the depth of the sequencing was sufficient to accurately characterize both the bacterial and fungal communities in the samples. Bacterial richness (Chao1 and Ace) was greater than fungal richness in all samples (**Table 1**), but there were no significant differences between the treatments (*p* = 0.10; α = 0.05). According to Shannon’s and Simpson’s indices, the bacterial diversity was higher in the treatments RP and TSP added (*p* = 0.02; α = 0.05) when compared to the control (**Table 1**), which showed lower equitability. For fungi, the distribution of OTUs among species (equitability) was favored to a higher extent by the RP treatment (Simpson’s index = 0.96), followed by the control (0.92) and the TSP treatment (0.86) (**Table 1**).

**Table 1:** Read numbers obtained from sequencing, OTU number, richness estimators, and diversity indices of the P treatments for bacteria and fungi community, adjusted for 37,000 and 100,000 reads, respectively.

				Richness estimator		Diversity index	
Group	Treatment^1^	N° reads^2^	N° OTU^3^	Chao1^4^	ACE^5^	Shannon^6,7^	Simpson^8^
Bacteria	Control	84,224 ± 23,034	1258 ± 140 a	1329 ± 145 a	1315 ± 140 a	6.4 ± 0.4 b	0.92 ± 0.03 b
	RP	77,972 ± 19,846	1441 ± 128 a	1513 ± 116 a	1501 ± 116 a	7.6 ± 0.35 a	0.98 ± 0.01 a
	TSP	60,211 ± 12,328	1463 ± 88 a	1536 ± 79 a	1519 ± 82 a	8.3 ± 0.03 a	0.99 ± 0.01 a
Fungi	Control	257,511 ± 97,148	628 ± 44 a	649 ± 40 a	652 ± 42 a	5.4 ± 0.4 a	0.92 ± 0.03 a
	RP	269,627 ± 56,806	724 ± 84 a	759 ± 80 a	756 ± 83 a	6.1 ± 0.1 a	0.96 ± 0.01 a
	TSP	249,197 ± 53,537	658 ± 55 a	696 ± 54 a	696 ± 53 a	5.2 ± 0.8 a	0.86 ± 0.01 a

*^1^Treatments: control, without added P; RP, rock phosphate added; TSP, triple superphosphate added. ^2^Mean and standard error of the read numbers for triplicates in each condition. ^3^Analysis of variance adjusted to the negative binomial distribution followed of the contrast test at 5% for bacteria and fungi community. ^4,5,6,8^Analysis of variance followed by a Scott Knott test at 5% for bacteria and fungi. ^7^Analysis of variance using distribution of Poisson followed of the contrast test at 5% for fungi, according to the Shannon index. Means with the same letter is equal according to Scott Knott test or Contrast test at 5%.*

**FIGURE 1:**
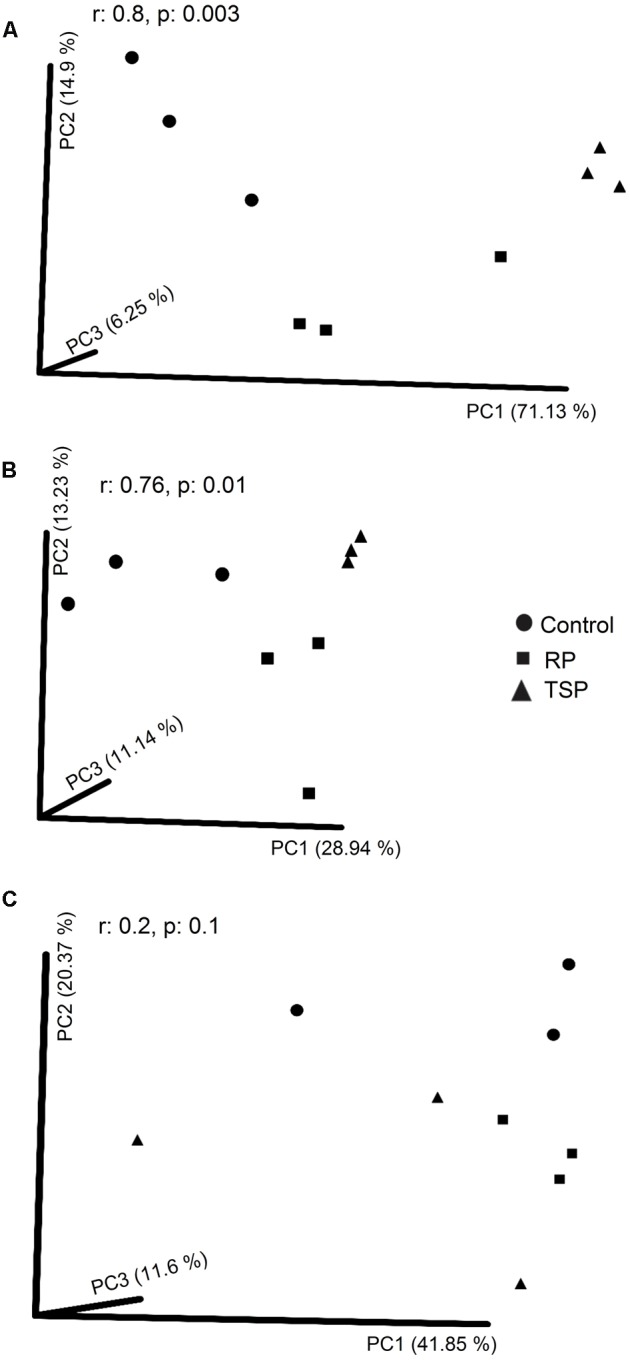
Principal coordinate analysis (PCoA) of the bacterial and fungal communities of the maize rhizosphere grown without added P (control), with rock phosphate (RP), and with triple superphosphate (TSP). Analysis of similarity (ANOSIM) was made between among the P treatments. **(A)** Clustering similarity of bacteria using weighted UniFrac and **(B)** using unweighted UniFrac. **(C)** Clustering similarity of the fungal community using the Bray–Curtis metric.

We used the distance matrix of weighted UniFrac (**Figure 1A**) and found a clustering of the bacterial communities into three groups, depending on the P treatments. However, the transformations based on the unweighted UniFrac distance showed a reduction in variance related to the P treatments (**Figure 1B**). For fungi, there was no separation of the samples at different P fertilization treatments using Bray–Curtis in the PCoA (**Figure 1C**). However, T-RFLP allowed grouping the samples (*p* = 0.003) depending on the given P treatments for the AMF (**Supplementary Figure S2**).

**FIGURE 2:**
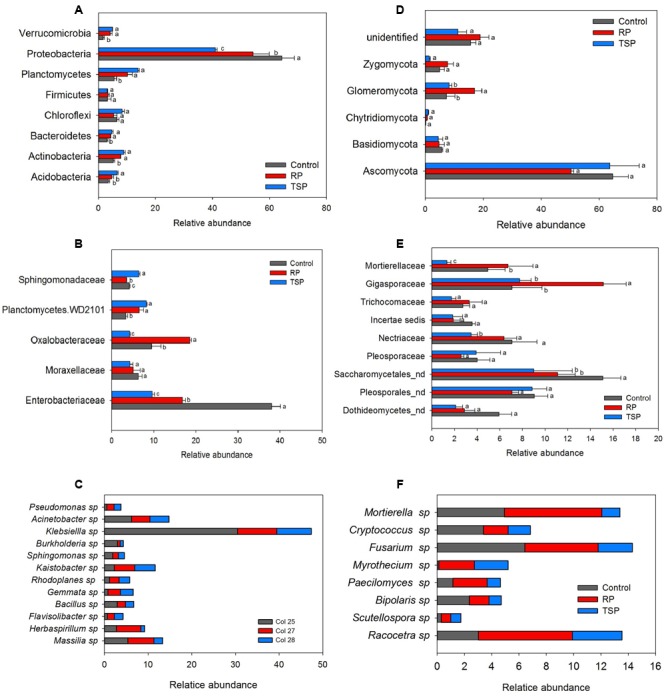
Relative abundance of **(A)** bacterial phyla, **(B)** families, and **(C)** genera. **(D)** Fungal phyla, **(E)** families, and **(F)** genera. Taxa occurring in at least one condition with an abundance greater than 3% were considered, and variance analysis was performed between P treatments, followed by Scott Knott test at 5%. For each taxon, means with the same letter are not significantly different. Control, treatment without added P; RP, treatment with rock phosphate; TSP, treatment with triple superphosphate; nd, identification was not possible at the family level.

**FIGURE 3:**
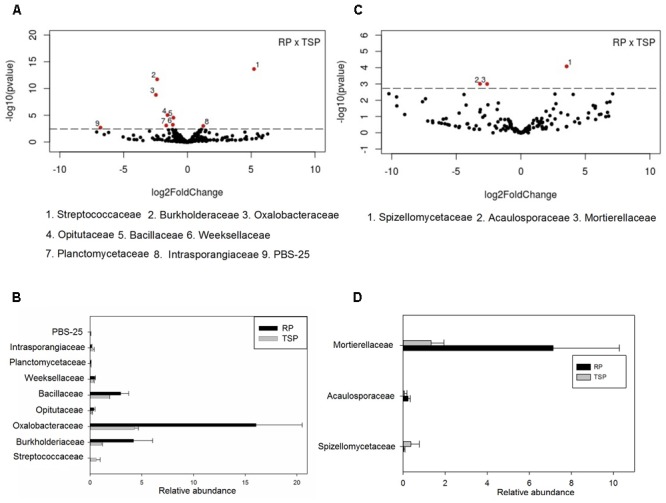
Significance analysis of the relative abundance of the taxa until family level (threshold of 0.1%) for bacterial **(A)** and fungal **(C)** communities using the edgeR analysis. In the volcano plot (**A** and **C**), the taxa that differ between RP and TSP treatments are showed above dashed line of the *y*-axis, corresponding to 0.05 of *p*-value and *x*-axis represents the fold changes in relative abundance (log2) of the taxa between treatments. Relative abundance only of the significant taxa in the edgeR analysis (volcano plot) for bacteria community **(B)** and **(D)** fungi community of the RP compared to TSP.

In general, differences in the abundance of bacterial community taxa were observed between P treatments (**Figure 2**). Regarding RP fertilized soil, the most abundant phylum was Proteobacteria (52%), followed by Planctomycetes (11%), Actinobacteria (7.8%) and Chloroflexi (5.8%) (**Figure 2A**). In the TSP, Proteobacteria (41%) was also the most abundant, followed by Planctomycetes (13.6%), Actinobacteria (8.6%), Chloroflexi (8.5%) and Acidobacteria (6.4%). On the control, the phylum Proteobacteria corresponded to 64.7% of the reads, followed by Chloroflexi with 6.2%, Planctomycetes with 5.6% and Actinobacteria with 5.4%. The major alteration corresponded to the decrease on the abundance of Proteobacteria with the use of TSP and RP in relation to control. For the other phyla, with exception for Firmicutes and Chloroflexi, it was observed an inverse effect: the relative abundance increased with the use of these P sources. **Figure 2B** summarizes the alterations observed in the bacterial community at family level. The dominant families were Enterobacteriaceae and Oxalobacteraceae. In the RP fertilized soil, there was a predominance of Oxalobacteraceae compared to the control and TSP treatments. Enterobacteriaceae was stimulated on the control and RP treatments (**Figure 2B**). The *Massilia* and *Herbaspirillum* genera were the most abundant of Oxalobacteraceae in the RP soil (**Figure 2C**). In addition, *Klebsiella* genus was the most abundant OTU found in the bacterial community, indicating its contribution to the differentiation of samples between P sources. The main alterations between the bacterial and fungal communities on the maize rhizosphere cultivated with TSP and RP were further explored by the Volcano plot (**Figure 3A**) that shown a general perspective of the OTUs’ fold changes between treatments according to the significance analysis of the relative abundance of the taxa using edgeR analysis. It was also observed predominance of Oxalobacteraceae in the RP fertilized soil compared to the TSP added soil, following by Burkholderiaceae and Bacillaceae (**Figure 3B**), comprised by *Burkholderia* and *Bacillus* genera (**Figure 2C**).

For the fungi, variation of the taxa abundance was also observed as a function of the P source. Ascomycota dominated in all experimental setup systems (**Figure 2D**). The second most abundant phylum was Glomeromycota, followed by Basidiomycota, Zygomycota, and Chytridiomycota. In the RP fertilized soil, the Glomeromycota OTUs were enriched in comparison to other treatments (**Figure 2D**), and Gigasporaceae was the dominant family in this niche (**Figure 2E**). *Scutellospora* and *Racocetra* were the most representative genera of this family (**Figure 2F**). Other families were abundant in the RP treatment in relation to TSP, according to edgeR analysis showed in the Volcano Plot (**Figures 3C,D**), such as Mortierellaceae and Acaulosporaceae, which showed *Mortierella* (**Figure 2F**) and *Acaulospora* (not showed data) as representative genera, respectively. Moreover, Saccharomycetales predominated in the control soil.

### Soil P Content and Productivity of the Maize are Related to the Microbial Community

**FIGURE 4:**
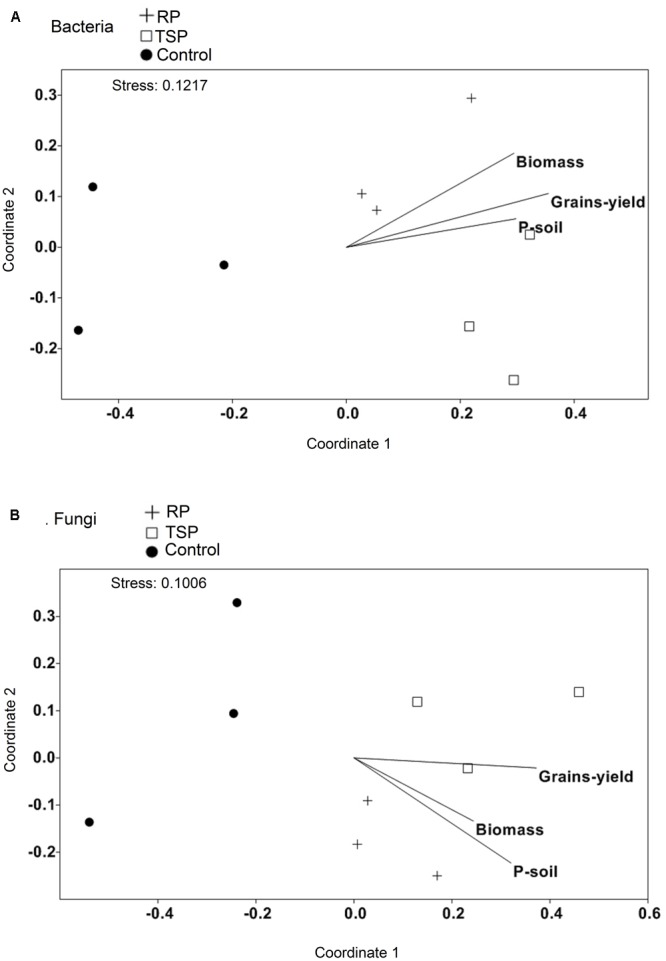
Non-metric multidimensional scaling (NMDS) of the composition of the **(A)** bacterial community and **(B)** the fungal community using a Rho metric. The samples were plotted with fitted environmental variables (biomass of the plant, grain yield, and P in the soil at the end of the experiment). Arrows indicate the direction of the correlation with the microbial community. For community composition, an OTU richness and phyla abundance with more than 1% of relative abundance were used. Treatments: soil without added P (control), soil treated with rock phosphate (RP), and soil with added triple superphosphate (TSP).

We used NMDS analysis to assess whether any parameters such as plant biomass, maize productivity, and P content on the soil (Supplementary Table S3) could be associated with the taxonomic variation of the microbial communities amidst P treatments (**Figure 4**). The formation of two groups was observed, one composed of the samples of the RP- and TSP-fertilized soils and the control soil, suggesting that the bacterial and fungal communities profile was altered in the control soil. For RP and TSP treatments, we observed positive correlation of the samples with grain yield, maize biomass and P content in the soil. Additionally, the values of all these parameters for the control decreased in relation to the RP- or TSP-fertilized soil (Supplementary Table S3).

## Discussion

We used a metataxonomic approach to characterize the structure of the microbial community at maize rhizosphere under different P sources. The P fertilization type modified the taxa profile of the microbial community of maize rhizosphere (**Figure 1**). The abundance of OTUs from bacteria were more affected by P sources than richness, as noted in the separation of the bacterial community by weighted UniFrac, which considers phylogenetic affiliations and OTUs abundance (**Figure 1A**). These results are different from the unweighted UniFrac (**Figure 1B**), which is sensitive only to the presence or absence of taxa, but not to OTUs abundance. Additionally, the Shannon and Simpson indices, which consider OTUs abundance, showed significantly higher values for P added samples (RP and TSP) in relation to the control for bacteria (**Table 1**). These results are different from previous studies that described a increase of the bacterial diversity in the P unfertilized soil (da Silva and Nahas, 2002; Toljander et al., 2008). It is possible that the effects of P sources on bacterial diversity are variable and are likely to be site-dependent. Moreover, such effects can be somehow associated to the soil and rhizosphere microbiota capacity of recovery from environmental perturbations, mainly after long periods of exposition to such condition. Factors like soil chemistry, plant genotype, management techniques, and plant growth stage also has modified the bacterial community associated with the maize (Castellanos et al., 2009; Cavaglieri et al., 2009; Aira et al., 2010; Li et al., 2014).

We also evaluated which taxa were enriched or decreased in the RP treatment in relation to the TSP treatment and the control. Proteobacteria was the dominant phylum in all P treatments (**Figure 2A**), and was found to be predominant in the microbial community of the maize rhizosphere (Chauhan et al., 2011; Peiffer et al., 2013; Li et al., 2014; Yang et al., 2017). Different taxa related to this phyla responded differently to P treatments. While Enterobacteriacea taxa (gammaproteobacteriacee) decreased with TSP and RP, Oxalobacteraceae and Burkholderiaceae (betaproteobacteria) increased with RP addition (**Figures 2B, 3B**). Bacillaceae (Firmicutes) showed significantly higher abundance in RP when compared to TSP-fertilized soil (**Figure 3B**). The taxa stimulated in response to cultivation using RP share similarity with those found in the *Phaseolus vulgaris* rhizosphere such as Oxalobacteraceae, Enterobacteriaceae, besides Actinobacteria, Comamonadaceae, Bradyrhizobacteriaceae, and Pseudomonodaceae (Trabelsi et al., 2017).

*Klebsiella* sp., the most abundant genera of Enterobacteriaceae (**Figure 2C**), has been frequently found in association with the maize crop (Brusetti et al., 2005; Roesch et al., 2007; Arruda et al., 2013), and it comprises bacterial species that are able to improve P release (Rajput et al., 2013; Walpola et al., 2014). Our group isolated *Klebsiella* from the rhizosphere and from the endophytic microbiota of maize cultivated in low P soil (Vieira et al., unpublished data). *Massilia* and *Herbaspirillum* are the most abundant genera of the Oxalobacteraceae family, and have already been described as P solubilizing bacteria (Estrada et al., 2013; Wang et al., 2016). *Burkholderia* sp. (Burkholderiaceae) and *Bacillus* sp. (Bacillaceae) are also reported to be effective RP solubilizers, both *in vitro* assays (Gomes et al., 2014; Ghosh et al., 2016) or inoculated in plants (Baig et al., 2014; Stephen et al., 2015; Wahid et al., 2016). Furthermore, *Burkholderia* and *Herbaspirillum* are both diazotrophs.

Regarding fungal community, although clustering related to P source were not significant on PCoA (**Figure 1C**), the AMF community was divided according to P treatments using T-RFLP analysis (**Supplementary Figure S2**). Additionally, the Glomeromycota phylum showed the most evident change between treatments, being the most abundant phylum on RP added soil (**Figure 2D**). Among the AMF families, Gigasporaceae was significantly more abundant on the RP added soil (**Figure 2E**). *Scutellospora* and *Racocetra* genera were predominant in this family (**Figure 2F**). Our research group has also found Gigasporaceae in the rhizosphere of maize cultivated in low P level soil, especially *Racocetra* sp. (unpublished data). Acaulosporaceae family also showed a significant increase in their abundance in the RP added soil when compared to soil with TSP (*p* < 0.05) (**Figures 3C,D**). P absorption by maize can be related to greater AMF presence on RP soil, since plant–AMF symbiosis supports P supply to plants on low P availability soils (Nouri et al., 2014). Thus, this indicates that these fungi may have contributed to maize productivity in RP treatment (Supplementary Table S3). *Mortierella* sp. also showed major abundance on RP-fertilized soil (**Figure 2F**), and was already reported as RP solubilizing fungi (Osorio and Habte, 2013; Vega et al., 2015). Moreover, synergistic effects of the inoculation of *Mortierella* sp. with AMF for plant P uptake and growth have been described (Zhang et al., 2011; Osorio and Habte, 2013).

Some studies have reported bacteria as symbionts of AMF hyphae. These were defined as “mycorrhiza helper bacteria,” which stimulate mycelial growth and/or contribute to inhibition of competitors and antagonists (Frey-Klett et al., 2007; Offre et al., 2008; Scheublin et al., 2010). Varied strains found in this work show symbiose with AMF. *Massilia* sp., for example, was reported by Cruz et al. (2008) to be associated to spores of AMF. *Burkholderia* and *Bacillus* genera were also reported as AMF symbionts from hyphosphera (Levy et al., 2003; Frey-Klett et al., 2007), and some *Burkholderia* strains isolated from AMF hyphae showed RP solubilization capacity (Taktek et al., 2015, 2017; Trabelsi et al., 2017). Thus, this result suggests that the high abundance of *Massilia* (Oxalobacteraceae), *Burkholderia* (Burkholderiaceae) and *Bacillus* (Bacillaceae) in the RP-fertilized soil could be associated to the greater abundance of AMF in this treatment when compared to the others (**Figures 2C, 3B**). Moreover, some bacterial genera enriched in the RP treatment have been described as auxin producers including *Herbaspirillum* spp. (Brusamarello-Santos et al., 2012), *Burkholderia* sp. and *Bacillus* sp. (Galdiano et al., 2011). This phytohormone is known to stimulate root growth that can also help P uptake by the plant once it increases its absorption surface area.

A lower AMF abundance in the TSP supplemented soil was expected since there is a significant decrease in the percentage of AMF colonization in the soil with high P level available (Smith et al., 1992). However, there was also a lower AMF abundance in the soil with no P addition, possibly due to the occurrence of P competition between the AMF hyphae and roots in the rhizosphere (Smith et al., 2011). Some studies have demonstrated that plants cultivated in very low P doses have decreased AMF colonization (Amijee et al., 1989; Koide and Li, 1990).

Our results are consistent with the long-term selection of P solubilizing microbial community. These microorganisms could be contributing to an increase on the P content in the RP-fertilized soil at the end of the 3 years of cultivation and to the facilitated P absorption by plants. This is evidenced by the maize plants growth profile cultivated in soil added RP, a low solubility phosphorus source, that was comparable to that of the plants cultivated in the TSP-added soil (**Figure 4** and Supplementary Table S3). However, we do not neglect the plant’s ability in acquiring nutrients by itself and the effects of physical–chemical processes over the 3-year cultivation, which may also contribute for soil P dynamics and consequently help its release in RP-fertilized soil (Goedert and Lobato, 1980; Coutinho et al., 1991). While it is not possible to infer cause-effect relationship in microbiota-phosphate acquisition, our study points to a few candidate groups possibly involved RP solubilization, which could be further evaluated as inoculants in greenhouse and field studies.

## Author Contributions

US, SC-O, CO, EG, and VD designed the experiment. US, JM, and UL obtained and processed the data. US, JM, LL, and DM analyzed the data. US and VD wrote the paper with contribution of all co-authors.

## Conflict of Interest Statement

The authors declare that the research was conducted in the absence of any commercial or financial relationships that could be construed as a potential conflict of interest.

## Supplementary Material

FIGURE S1 Rarefaction curves of **(A)** bacteria and **(C)** fungi using QIIME software were calculated according observed OTU number in the treatment means. Control, without added P; RP, rock phosphate addition; TSP, addition of triple superphosphate. Furthermore, 0.99 of Good Coverage values from 10,000 reads for **(B)** bacteria and 20,000 for **(D)** fungi was observed in all treatments.

FIGURE S2 Community of mycorrhizal fungi detected in the soil fertilized with RP, TSP, and without the addition of P (control). Non-metric multidimensional scaling (NMDS) was based on the matrix of Jaccard and it was performed variance analysis of the distance matrix using the multivariate permutation test (Adonis).
