# Supplementary figures and images for: Long-Term Rock Phosphate Fertilization Impacts the Microbial Communities of Maize Rhizosphere
Source: Front Microbiol. 2017 Jul 11;8:1266. doi: 10.3389/fmicb.2017.01266 (PMC5504191; doi:10.3389/fmicb.2017.01266)

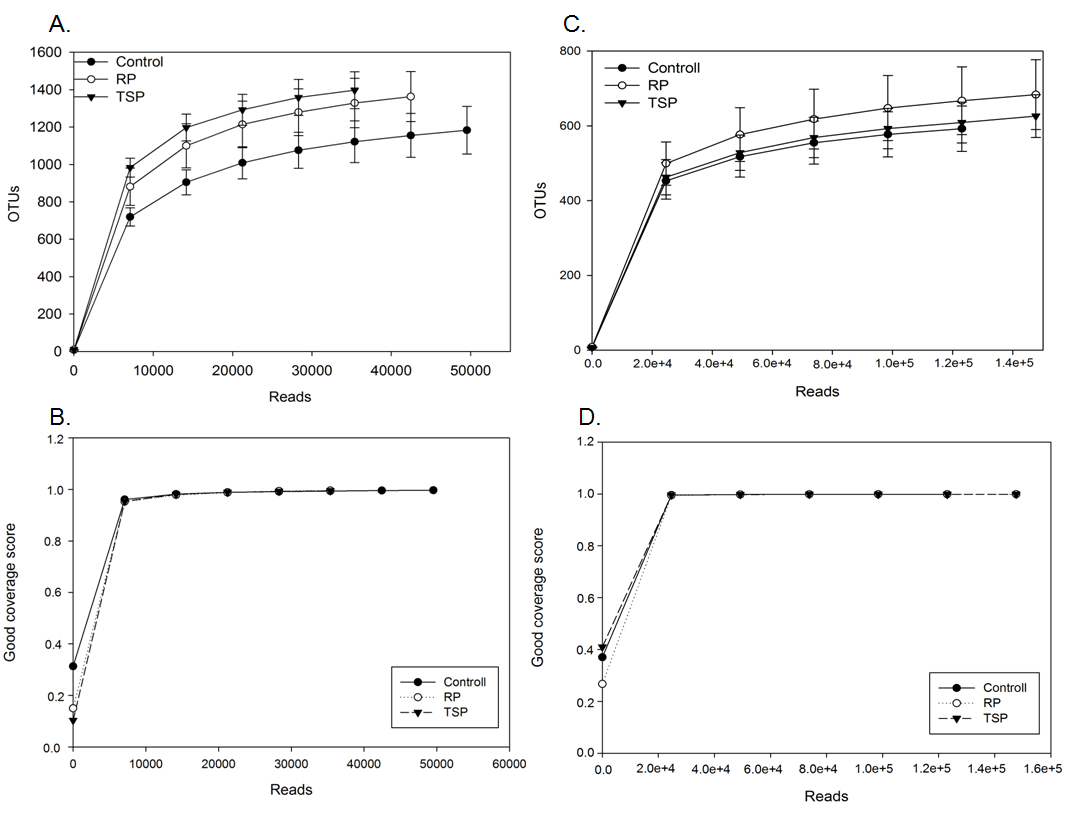

Supplement: FIGURE S1 — Rarefaction curves of (A) bacteria and (C) fungi using QIIME software were calculated according observed OTU number in the treatment means. Control, without added P; RP, rock phosphate addition; TSP, addition of triple superphosphate. Furthermore, 0.99 of Good Coverage values from 10,000 reads for (B) bacteria and 20,000 for (D) fungi was observed in all treatments. [file Image_1.TIFF]

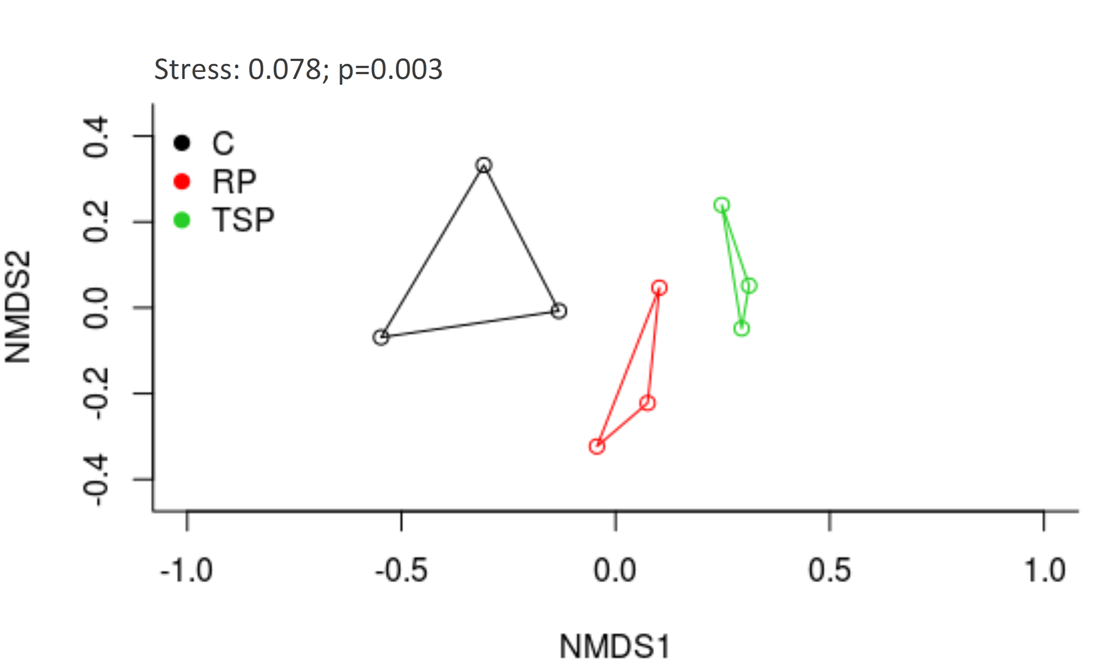

Supplement: FIGURE S2 — Community of mycorrhizal fungi detected in the soil fertilized with RP, TSP, and without the addition of P (control). Non-metric multidimensional scaling (NMDS) was based on the matrix of Jaccard and it was performed variance analysis of the distance matrix using the multivariate permutation test (Adonis). [file Image_2.TIF]
