# Supplementary material for: Long-Term Rock Phosphate Fertilization Impacts the Microbial Communities of Maize Rhizosphere
Source: Front Microbiol. 2017 Jul 11;8:1266. doi: 10.3389/fmicb.2017.01266 (PMC5504191; doi:10.3389/fmicb.2017.01266)
Supplement: Supplementary file 3 [file Table_1.DOC]

**Supporting information**

**Table S1.** Characteristics of the Oxisol agricultural soil of the Brazilian savanna biome

(Cerrado) and of the rock phosphate.

| **Soil physical and chemical characteristics*** | | | |
| --- | --- | --- | --- |
|  | | **0 – 20 cm** | **20 – 40 cm** |
| pH (H_2_O) | | 5.80 | 5.40 |
| P (mg dm^3^)** | | 3.94 | 0.68 |
| ^1^MO (^2^dag Kg^-1^) | | 3.91 | 3.15 |
| C (%) | | 2.33 | 1.83 |
| ^3^CEC (^4^cmoc dm^3^) | | 10.57 | 9.23 |
| Fe (mg dm^3^) | | 27.00 | 30.00 |
| Zn (mg dm^3^) | | 2.01 | 0.40 |
| Al (cmoc dm^3^) | | 0.02 | 0.36 |
| Ca (cmoc dm^3^) | | 2.11 | 0.79 |
| Mg (cmoc dm^3^) | | 0.57 | 0.11 |
| K (mg dm^3^)** | | 20.00 | 9.00 |
| Cu (mg dm^3^) | | 1.00 | 1.04 |
| Mn (mg dm^3^) | | 7.20 | 4.30 |
| **Rock phosphate characteristics ^***^** | | | |
| P_2_O_5_ (%) | Total | | 36 |
|  | Citric acid | | 5 |
|  | Neutral ammonium citrate | | 2 |

^*^ A soil/water ratio of 1:2.5 (w/v) was used.

^**^ P and K were quantified by Mehlich (HCl 0.05N and H_2_SO_4_ 0.025N)

^***^ Total P content of the rock phosphate of Araxá and its solubility in 2 % citric acid or neutral ammonium citrate.

^1^. Organic matter (MO): Carbon x 1.72.

^2^ Decagrams (dag)

^3^ Cation exchange capacity (CEC)

^4^ One hundred mols of charge per kilogram (cmoc)
