# Supplementary material for: Long-Term Rock Phosphate Fertilization Impacts the Microbial Communities of Maize Rhizosphere
Source: Front Microbiol. 2017 Jul 11;8:1266. doi: 10.3389/fmicb.2017.01266 (PMC5504191; doi:10.3389/fmicb.2017.01266)
Supplement: Supplementary file 4 [file Table_2.DOC]

**Supporting information**

**Table S2:** Primers used in the metataxonomic and T-RFLP experiments

| Primer | Target region | Sequence | Reference |
| --- | --- | --- | --- |
| 341F | 16 S rRNA gene,  V3 and V4 | 5′ CCTACGGGNGGCWGCAG 3′ | Klindworth *et al.* (2013) |
| 806 R | 16 S rRNA gene,  V3 and V4 | 5′ GGACTACHVGGGTWTCTAAT 3′ | Klindworth *et al.* (2013) |
| ITS3_KYO1F | ITS2 | 5’ AHCGATGAAGAACRYAG 3′ | Toju *et al.* (2012) |
| ITS4_KYO1R | ITS2 | 5' TCCTCCGCTTWTTGWTWTGC 3' | Toju *et al.* (2012) |
| LR1 | 28S rRNA gene | 5'GCATATCAATAAGCGGAGGA 3' | Trouvelot *et al.* (1999) |
| FLR2 | 28S rRNA gene | 5'GTCGTTTAAAGCCATTACGTC 3' | Trouvelot *et al.* (1999) |
| FLR3 | 28S rRNA gene,  D1 and D2 domains | 5' TTGAAAGGGAAACGATTGAAGT 3' | Gollotte, Van Tuinen, and Atkinson (2004) |
| FLR4 | 28S rRNA gene, D2 domain | 5' TACGTCAACATCCTTAACGAA 3' | Gollotte, Van Tuinen, and Atkinson (2004) |
