# Supplementary material for: Long-Term Rock Phosphate Fertilization Impacts the Microbial Communities of Maize Rhizosphere
Source: Front Microbiol. 2017 Jul 11;8:1266. doi: 10.3389/fmicb.2017.01266 (PMC5504191; doi:10.3389/fmicb.2017.01266)
Supplement: Supplementary file 5 [file Table_3.DOC]

**Supporting information**

**Table S3.** Growth parameters (biomass of the plant per hectare), productivity (grain yield per hectare) and nutrient content in the plant and in the grain per hectare: phosphorus (P), potassium (K), calcium (Ca), magnesium (Mg), sulfur (S), copper (Cu), iron (Fe) and zinc (Zn). The same letters between treatments indicate that there was no significant difference at 0.05 % by Scott Knott test.

| **Treat.^*^** | **Plant (Kg ha^-1^)** | | | | | | | | | **Biomass plant** | **P soil ^**^** |
| --- | --- | --- | --- | --- | --- | --- | --- | --- | --- | --- | --- |
|  | P | K | Ca | Mg | S | Cu | Fe | Mn | Zn | (Kg ha^-1^) | (mg dm^-3^) |
| **Control** | 0.86 b | 9.70 a | 13.6 a | 7.42 b | 2.74 a | 0.02 a | 1.02 a | 0.18 a | 0.18 a | 3160 b | 1.87 b |
| **RP** | 1.52 a | 14.0 a | 18.4 a | 13.1 a | 3.48 a | 0.02 a | 1.06 a | 0.22 a | 0.20 a | 5137 a | 17.56 a |
| **TSP** | 1.26 a | 15.1 a | 22.6 a | 11.8 a | 3.38 a | 0.02 a | 2.18 a | 0.26 a | 0.16 a | 4918 a | 14.61 a |
|  | **Grain (Kg ha ^-1^)** | | | | | | | | | **Grain yield**  **(Kg ha^-1^)** | |
| **Control** | 3.60 b | 6.62 b | 0.20 b | 1.80 b | 3.46 b | 4.26 b | 0.04 b | 0.00 b | 0.04 b | 2638 b | |
| **RP** | 10.5 a | 18.7 a | 0.34 a | 4.30 a | 7.90 a | 8.94 a | 0.06 a | 0.02 a | 0.12 a | 6802 a | |
| **TSP** | 9.40 a | 16.9 a | 0.32 a | 4.10 a | 7.52 a | 10.1 a | 0.08 a | 0.02 a | 0.08 a | 6896 a | |

^*^ Treatments: soil without added P (control), soil treated with rock phosphate (RP) and soil with added triple superphosphate (TSP).

^**^ Phosphurus in the soil was quantified by Mehlich (HCl 0.05N and H_2_SO_4_ 0.025N).
